# Vamorolone: a novel metabolism resistant steroid that suppresses joint destruction in chronic polyarthritis with reduced systemic side effects

**DOI:** 10.1093/rheumatology/keaf129

**Published:** 2025-04-02

**Authors:** Ana Crastin, Arjan Shanker, Michael S Sagmeister, Angela Taylor, Gareth G Lavery, Karim Raza, Rowan S Hardy

**Affiliations:** Department of Biomedical Sciences, University of Birmingham, Birmingham, UK; Department of Biomedical Sciences, University of Birmingham, Birmingham, UK; Department of Metabolism and Systems Science, University of Birmingham, Birmingham, UK; Department of Metabolism and Systems Science, University of Birmingham, Birmingham, UK; Department of Biosciences, Centre for Systems Health and Integrated Metabolic Research, Nottingham Trent University, Nottingham, UK; Department of Inflammation and Aging, University of Birmingham, Birmingham, UK; Sandwell and West Birmingham NHS Trust, Birmingham, UK; Department of Biomedical Sciences, University of Birmingham, Birmingham, UK; Department of Metabolism and Systems Science, University of Birmingham, Birmingham, UK; MRC Arthritis Research UK Centre for Musculoskeletal Ageing Research, University of Birmingham, Birmingham, UK

**Keywords:** vamorolone, glucocorticoid, rheumatoid arthritis, prednisolone, steroid metabolism, polyarthritis, bone loss, muscle wasting

## Abstract

**Objectives:**

Vamorolone, a dissociated steroidal compound with reduced side effects, offers a promising alternative to traditional glucocorticoids for inflammatory diseases. Unlike conventional glucocorticoids, vamorolone lacks the hydroxyl or ketone groups required for metabolism by 11β-hydroxysteroid dehydrogenase type 1 (11β-HSD1), a key enzyme that modulates glucocorticoid activity. This study investigates vamorolone’s resistance to 11β-HSD1 metabolism and assesses its therapeutic efficacy in the murine tumour necros factor-alpha-overexpressing (TNFtg) model of polyarthritis.

**Methods:**

11β-HSD1 metabolism and action were examined in Hs68 and primary leucocyte culture. Vamorolone 20 mg/kg/day, prednisolone (standard of care) or vehicle were administered by gavage to TNFtg or TNFtg 11β-HSD1 knock-out (TNFtg^11BKOKO^) animals. Body weight and disease severity were scored daily, and markers of inflammation, joint destruction and side effects assessed at day 56 of age.

**Results:**

Vamorolone was entirely resistant to 11β-HSD1 metabolism *in vitro*. Vamorolone demonstrated comparable anti-inflammatory actions in TNFtg mice, with a comparable reduction in joint inflammation, serum interleukin-6 (IL-6) and synovitis relative to prednisolone. However, vamorolone-treated mice did not experience typical glucocorticoid side effects, including adrenal atrophy, body weight reduction, muscle wasting or inhibition of anabolic bone metabolism. These benefits persisted in 11β-HSD1 knockout mice, indicating that the efficacy of vamorolone is largely independent of 11β-HSD1 metabolism.

**Conclusion:**

The findings suggest that at the effective anti-inflammatory dose examined in this study, vamorolone possesses a reduced profile of deleterious systemic effects relative to prednisolone. Whilst highlighting its potential for broader clinical application in inflammatory conditions, it remains unclear whether these side effects would remain mild at markedly higher doses.

## Graphical abstract

**Figure:**
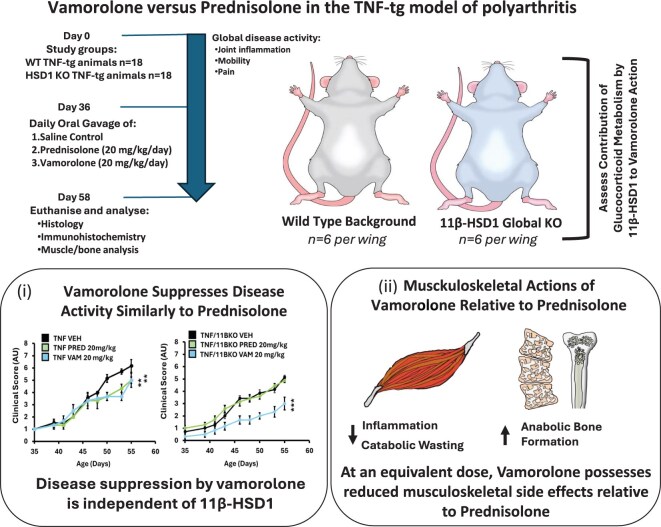

Rheumatology key messages

- The glucocorticoid vamorolone is resistant to cellular metabolism and amplification by the enzyme 11β-HSD1.
- It reduces inflammation and joint damage in models of polyarthritis comparably to prednisolone.
- In the TNFtg arthritis model, vamorolone did not elicit glucocorticoid-induced musculoskeletal side-effects.

## Introduction

**Figure 1.**
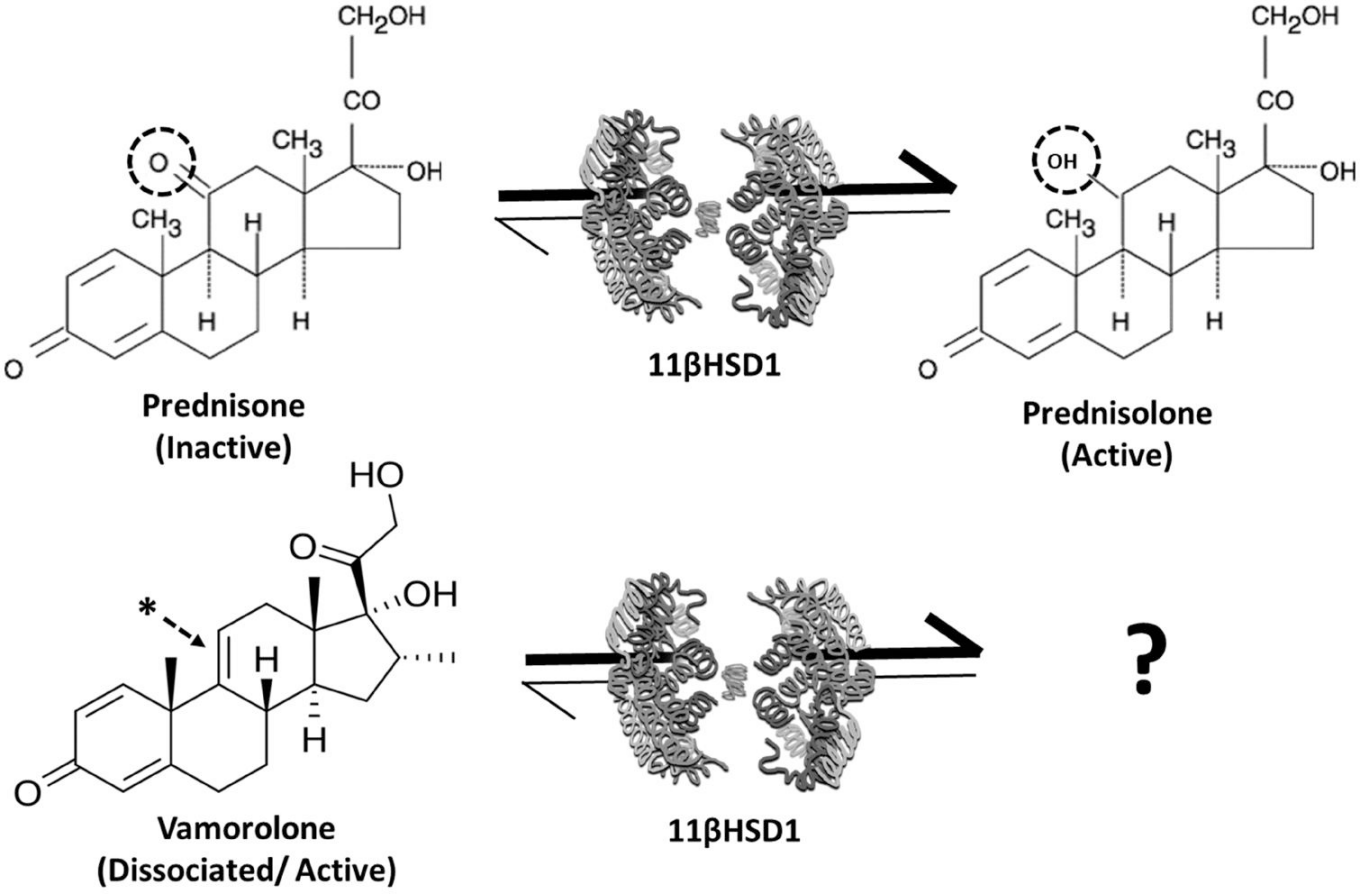
Vamorolone metabolism. Schematic depicting the conversion of prednisone to prednisolone by the enzyme 11β-HSD1. Oxoreductase activity favours activation of prednisolone from the inactive precursor prednisone, with the conversion of the ketone at C11 to a hydroxyl group (dotted circle). Vamorolone possess a double bond between C9 and C11 (denoted at the *), removing the classical site for 11β-HSD1 metabolism. 11β-HSD1: 11β-hydroxysteroid dehydrogenase type 1

Glucocorticoids such as prednisolone are prescribed to over 1% of the global population annually and play a pivotal role in the management of a diverse array of chronic inflammatory diseases such as RA, asthma and IBD [1, 2]. Approximately 70% of patients develop adverse effects, including sarcopenia, osteoporosis, hypertension and insulin resistance [3]. The enzyme 11β-hydroxysteroid dehydrogenase type 1 (11β-HSD1) favours the pre-receptor activation of glucocorticoids such as cortisol and prednisolone, from inactive circulating precursors (Fig. 1) [4]. This is mediated by the reduction of a ketone at the carbon-11 (C11) position of the steroid to a hydroxyl group, conveying increased affinity to the glucocorticoid receptor [5]. Within tissues such as liver, adipose, skeletal muscle and bone, 11β-HSD1 drives pre-receptor activation and amplification of glucocorticoid signalling, directly contributing to side effects such as insulin resistance, osteoporosis and muscle wasting [6–8].

Vamorolone is a dissociated steroidal anti-inflammatory drug effective in the treatment of patients with Duchenne muscular dystrophy that possesses a significantly reduced profile of undesirable side effects relative to traditional glucocorticoids [9–13]. Vamorolone has also shown significant promise in the suppression of disease activity in an array of disease models, including allergic asthma, IBD and CIA [13–16]. Vamorolone shares structural homology with glucocorticoids such as prednisolone, but possesses a double bond between C9 and C11, preventing the formation of a functional hydroxyl, or ketone group at C11 (Fig. 1) [17, 18]. This change conveys the dissociated properties of vamorolone at the glucocorticoid receptor and removes the traditional targets required for metabolism and amplification by 11β-HSD1. We have shown that this enzyme mediates many of the deleterious off-target actions of oral glucocorticoids [6, 8, 19, 20]. Whether the improved side effects profile reported for vamorolone reflects evasion of local metabolism and amplification by 11β-HSD1 at off target sites such as the adipose, muscle and bone have yet to be examined. To determine this, we utilized *in vitro* enzyme models to monitor 11β-HSD1 metabolism of vamorolone, before using animals with global transgenic deletion 11β-HSD1 to assess its contribution to the actions of vamorolone in the TNFtg model of polyarthritis.

## Methods

### Models of polyarthritis

Procedures were performed under guidelines by the Animal (Scientific Procedures) Act 1986 in accordance with the project licence (P51102987) and approved by the Birmingham Ethical Review Subcommittee (BERSC). The TNFtg model of chronic inflammatory polyarthritis, obtained courtesy of Professor George Kollias (BSRC Fleming, Athens), was maintained on a C57BL/6 background and compared with WT littermates [21]. At day 36 of age, at the first onset of measurable polyarthritis, male TNFtg and TNFtg^HSD1KO^ global knockout mice received either 20 mg/kg/day of vamorolone suspension in sterile water or 20 mg/kg/day Prednisolone suspension in sterile water through oral gavage for 21 days. Mice were scored for global disease activity, joint inflammation, mobility and pain as previously described [22, 23]. At day 56 serum was collected by cardiac puncture and tissues excised for analysis.

### Histological staining and immunohistochemistry

Front paws were dissected and fixed in 4% paraformaldehyde, paraffin embedded and sectioned at 5 µm. Sections were stained with haematoxylin and eosin for histological analysis. Articular synovitis, pannus size at the humerus/ulna elbow joint interface and cartilage area at the articular surface of the tibia were determined using ImageJ software as previously reported [8, 22].

Quantification of *F4/80*-positive cells in a 50-µm^2^ area was determined by immunohistochemistry. F4/80 (anti-Mouse) monoclonal antibody (Invitrogen, Thermo Fisher Scientific, Birmingham, UK, 14-4801-85, 1:100) was used to detect myeloid populations and detected using the ImmPRESS HRP Kit (Goat Anti-Rat/Mouse IgG) (Vector labs, Kirtlington, UK, MP-744) as per manufacturer instructions.

### Histological analysis of muscle

Murine quadriceps muscles were embedded in paraffin and cut to 10-µm sections for histology. Samples were stained with haematoxylin and eosin prior to quantitative analysis of fibre size distribution using ImageJ software. Measurements were taken in three 200-µm^2^ regions of the vastus medialis for six mice per group, and reported in µm^2^.

### Primary macrophage culture

Macrophages were generated using monocytes obtained from blood cones from healthy fully anonymized donors, from the NHS Blood and Transplant Centre, Birmingham, under University of Birmingham Ethics Committee approval (ERN_14-0446). CD14+ monocytes were isolated from blood using the RosetteSep™ Human Monocyte Enrichment Cocktail (Stem Cell, Cambridge, UK), as per manufacturer’s guidelines. Monocytes were differentiated into M0 unpolarized macrophages by culturing with 10% fetal bovine serum, 1% penicillin–streptomycin, and M-CSF 20 ng/ml for 6 days. M1-like polarization was achieved by culturing with either IFNγ/TNFα (10 ng/ml) for 24 h. M1-like macrophages were treated with either culture media (control), dexamethasone at 100 nM/l or vamorolone at 1000 nM/l for 24 h.

### HS68 culture

Human foreskin fibroblast cell line (HS68) obtained from the European Collection of Authenticated Cell Cultures (ECACC, Salisbury, UK) were cultured in DMEM (Gibco, Thermo Fisher Scientific, Birmingham, UK) supplemented with 10% fetal bovine serum and 1% penicillin–streptomycin.

### 11β-Hydroxysteroid reductase enzyme assay

Confluent cells were incubated with 1 ml DMEM medium, containing either 100 nmol/l cortisone (+ H^3^ labelled-tracer) or 100 nmol/l vamorolone (+ C^14^ labelled-tracer) with or without glycyrrhetinic acid (GE) at 1000 nM/l for 18 h. Steroids were extracted under dichloromethane and separated by thin-layer chromatography using ethanol:chloroform (8:92) as previously described [24]. Thin-layer chromatography plates were analysed using a Bioscan imaging detector (Bioscan, Washington, DC, USA) and fractional conversion calculated and normalized to total cells number. Results were expressed as pmol product/h/million cells.

### mRNA extraction from mouse tissues

Quadriceps and flushed femur mRNA extracted using TRI reagent (AM9738, Thermofisher) as per manufacturers protocol. The RNA pellet was washed with 75% ethanol and resuspended in RNase-free water. Concentrations were determined by NanoDrop.

### Gene expression analysis

Gene expression was assessed by TaqMan^®^ Gene Expression Assays (ThermoFisher Scientific, Birmingham, UK) and reverse transcription (Multiscribe^TM^, ThermoFisher Scientific, Birmingham, UK) as per the manufacturer’s guidelines. Expression of genes was determined using species-specific probe sets by real-time PCR on an QuantStudio™ 5 Real-Time PCR System (Applied Biosystems, Warrington, UK) (Table 1). mRNA abundance was normalized to 18S. Data, obtained as Ct values and ΔCt determined (Ct target—Ct 18S or GAPDH), were expressed as arbitrary units (AU) using the following transformation: [AU = 1000 × (2^−ΔCt^)].

**Table 1.** Quantitative PCR primer probes

Target gene	Reference number
*18S*	431943E
*GILZ*	Hs00608272_m1
*TNFα*	Hs01113624_g1
*Tnfa*	Mm00443258
*Fbxo32*	Mm00499523
*Foxo1*	Mm00490672
*Mstn*	Mm01185221
*Bglap*	Mm03413826
*IL-6*	Mm00446190
*Trim63*	Mm01185221
*Il-1b*	Mm00434228
*Ifna*	Mm02525960
*Ifnb*	Mm00439552

### Analysis of P1NP, CTX, ACTH and IL-6 by ELISA

Serum was collected from mice by cardiac puncture under terminal anaesthetic. Briefly, whole blood was left at room temperature for 30 min prior to centrifugation for 20 min at 12 000 rpm. Serum was aspirated and stored at −80°C prior to analysis. Serum P1NP was determined using a commercially available sandwich ELISA (cat no: AC-33F1, Immunodiagnostic Systems, Tyne & Wear, UK) in accordance with the manufacturer’s instructions, and data expressed as ng/ml. Serum CTX-1 was determined using a commercially available sandwich ELISA (cat no: AC-06F1, Immunodiagnostic Systems, Tyne & Wear, UK) in accordance with the manufacturer’s instructions, and data expressed as units per microlitre. Production of mouse ACTH (adrenocorticotrophic hormone) and IL-6 in serum were assessed using ELISA. Mouse Coated ELISA Kits for ACTH and IL-6 (Invitrogen, ThermoFisher Scientific, Birmingham, UK) were performed as per manufacturer’s protocol on Precoated Quantikine^®^ plates.

### Statistical analysis

Data were analysed using IBM SPSS Statistics v28.0.1.0 (IBM Analytics, USA) and GraphPad Prism v5.03 and v9.5 (GraphPad Software, USA), with a *P*-value of <0.05 considered to be statistically significant. Normality of data was confirmed using the Shapiro–Wilk normality test. Data were analysed using Student’s *t*-test, or one-way ANOVA with *post hoc* Tukey’s test or two-way ANOVA with Tukey correction as appropriate. *In vitro* experiments were carried out with sample sizes of *n* ≥ 3, defined as independent primary cell cultures from different donors, unless stated otherwise in figure legend (*P ≤ 0.05, **P ≤ 0.01, ***P ≤ 0.001 and ****P ≤ 0.0001.

### Ethics

Procedures were performed under guidelines by the Animal (Scientific Procedures) Act 1986 in accordance with the project licence (P51102987) and approved by the Birmingham Ethical Review Subcommittee (BERSC).

## Results

### Vamorolone induces anti-inflammatory genes and reduces pro-inflammatory mediators *in vitro* but is resistant to 11β-HSD1 metabolism

**Figure 2.**
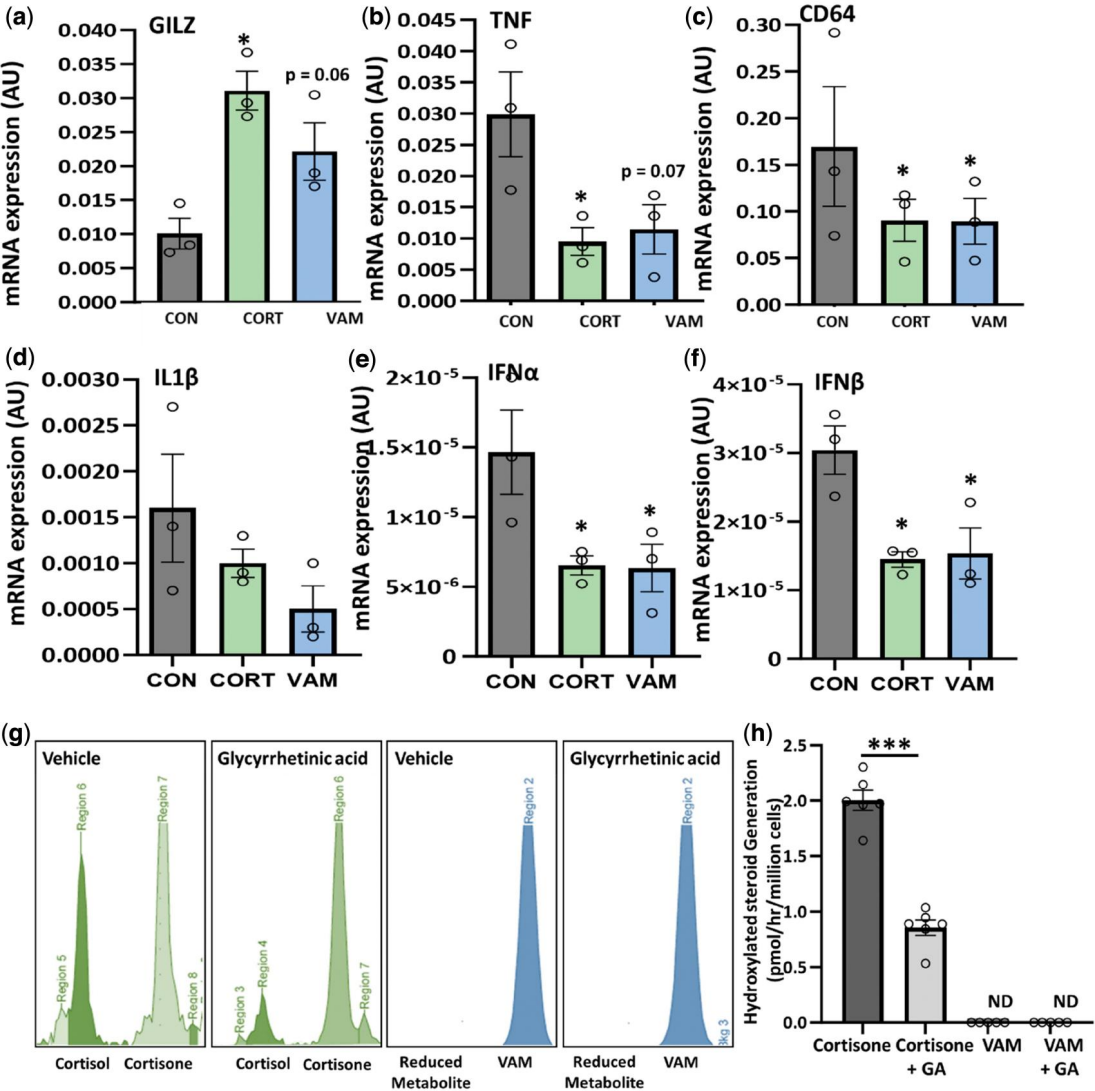
Vamorolone suppresses inflammation *in vitro* but is resistant to 11β-HSD1 metabolism. (**A**) *GiLZ*, (**B**) *TNFα*, (**C**) *CD64* (**D**) *IL1β* (**E**) *IFNα* and (**F**) *IFNβ* gene expression determined by quantitative RT-PCR in inflammatory polarized primary human macrophages treated with culture media (CON), 100 nM/l dexamethasone (DEX) and 1000 nM/l of vamorolone (VAM) for 24 h. (**G**) Tritiated cortisone to cortisol conversion in HS68 cells shown as two peaks generated by running thin-layer chromatography (vehicle); glycyrrhetinic acid partially inhibits tritiated cortisone to cortisol conversion whilst carbon-14-labelled vamorolone single peak (VAM) shown in vehicle and glycyrrhetinic acid–treated cells with absent reduced metabolite peak. (**H**) Hydroxylated steroid generation measured after 18 h treatment in HS68 cells treated with tritiated cortisone ± GE (glycyrrhetinic acid), carbon-14 vamorolone ± GE (glycyrrhetinic acid). Data are presented as mean ± s.e.m. of at least three primary cultures from three independent patient donors. Statistical significance was determined using one-way ANOVA with Dunnett’s multiple comparisons test (*P ≤ 0.05, **P ≤ 0.01, ***P ≤ 0.001). 11β-HSD1: 11β-hydroxysteroid dehydrogenase type 1

Inflammatory gene regulation was examined in macrophages in response to cortisol and vamorolone. Cortisol (100 nmol/l) significantly upregulated the expression of the pro-resolving mediator glucocorticoid-induced leucine zipper (*GILZ*) (3.2-fold; *P <* 0.05) and downregulated the expression of the potent pro-inflammatory mediator TNF-α (*TNFα*) (3.05-fold; *P <* 0.05) and the monocyte/macrophage marker cluster of differentiation 64 (*CD64*) (1.7-fold; *P <* 0.05) (Fig. 2A–C). Vamorolone (100 nmol/l) displayed a comparable, but partially attenuated capacity to regulate these genes with trends towards increased *GILZ* (2.1-fold; *P =* 0.06) and decreased *TNFα* (2.6-fold; *P =* 0.07) expression, and a significant suppression of *CD64* expression (1.8-fold; *P =* 0.05). Additional examination of glucocorticoid responsive inflammatory genes, including *IL-1β*, *IFNα* and *IFNβ*, revealed a similar pattern to that of *TNFα* and *CD64*, with a marked reduction in response to both cortisol and vamorolone, albeit falling short of significance for *IL-1*β (Fig. 2D–F). To assess the capacity of the glucocorticoid activating enzyme 11β-HSD1 to reduce the dissociated steroid vamorolone at position 11, oxo-reductase activity was measured in Hs68 fetal fibroblasts (Fig. 2D and E). 11β-HSD1 oxo-reductase activity was evident with marked cortisol activation from cortisone (2.03 pmol/h/million cells) (Fig. 2D and E). This was significantly suppressed (*P <* 0.001) following by the 11β-HSD inhibitor glycyrrhetinic acid (1 µmol/l) (Fig. 2D and E). Vamorolone showed no evidence of metabolism and conversion after incubation (Fig. 2D and E). The addition of GE did not influence this process. These data reveal that vamorolone influences inflammatory gene expression profile in macrophages similarly to cortisol, but was entirely resistant to canonical 11β-HSD1 metabolism.

### Vamorolone suppresses disease activity *in vivo* independently of 11β-HSD1

**Figure 3.**
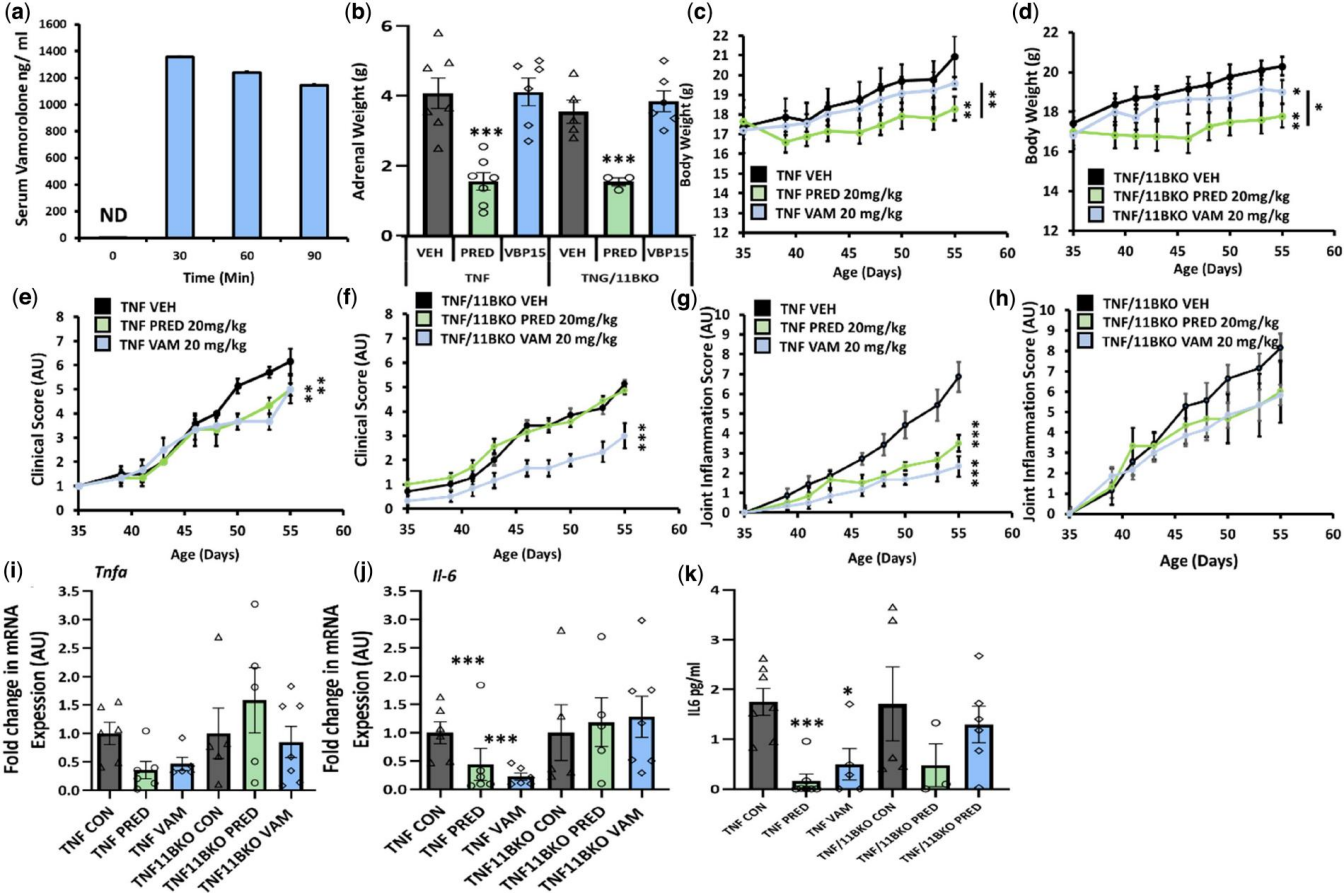
Vamorolone suppresses disease activity in vivo independently of 11β-HSD1. (**A**) Serum vamorolone measured using LC-MS at 30, 60, 90 min after oral gavage in three male mice. (**B**) Adrenal weight measured following 21 days of oral gavage with prednisolone (PRED) and vamorolone (VBP15) at 20 mg/kg in TNF (TNFtg) and TNF/11BKO (TNF transgenic 11bHSD1 knockout). (**C**) Body weight in TNF mice, (**D**) body weight in TNF/11BKO mice, (**E**) disease score in TNF mice, (**F**) disease score in TNF/11BKO mice, (**G**) inflammatory paw score in TNF mice and (**H**) inflammatory paw score in TNF/11BKO mice measured following 21 days of oral gavage with prednisolone (PRED) and vamorolone (VBP15) at 20 mg/kg. (**I**) Tnfa, (**J**) Il-6 gene expression and (**K**) IL-6 cytokine production measured following 21 days of oral gavage with prednisolone (PRED) and vamorolone (VBP15) at 20 mg/kg given to TNFtg (TNF) and TNF/11BKO (TNF transgenic 11βHSD1 knockout mice. Data are presented as mean ± s.e.m. of at least six mice per treatment group. Statistical significance was determined using one-way ANOVA with Tukey’s multiple comparisons test (*P ≤ 0.05, **P ≤ 0.01, ***P ≤ 0.001). 11β-HSD1: 11β-hydroxysteroid dehydrogenase type 1

The TNFtg murine model of polyarthritis on a wild type and 11β-HSD1 knockout (TNFtg^11BKO^) background was utilized to define its contribution to the immunomodulatory actions of vamorolone. Animals received either vamorolone or the prednisolone at 20 mg/kg/day by gavage from onset of joint inflammation to day 56. Vamorolone was elevated systemically after gavage, peaking at 1353 ± 58 ng/ml at 30 min and gradually declining over 90 min to 1140 + 36 ng/ml (Fig. 3A). In both TNFtg and TNFtg^11BKO^ animals, marked adrenal atrophy was evident in animals receiving prednisolone (TNFtg, 65.2%, *P <* 0.001; TNFtg^11BKO^, 57.2%, *P <* 0.001). In contrast, vamorolone did not elicit adrenal atrophy in either the TNFtg or TNFtg^11BKO^ cohorts (Fig. 3B).

Vehicle-treated TNFtg and TNFtg^11BKO^ animals receiving saline only, steadily gained weight over the duration of the study (Fig. 3B and C). In both the TNFtg and TNFtg^11BKO^ cohorts, prednisolone significantly reduced body weight relative to vehicle-treated controls upon completion of the study (TNFtg, 14.1%, *P <* 0.01; TNFtg^11BKO^, 12.4%, *P <* 0.01). No significant reduction in body weight relative to vehicle-treated controls was observed in TNFtg animals receiving vamorolone, whilst a less marked loss in bodyweight was apparent in TNFtg^11BKO^ animals receiving vamorolone (TNFtg^11BKO^, 6.5%, *P <* 0.05) (Fig. 3C and D). Bodyweights in prednisolone-treated animals were significantly lower at day 56 relative to vamorolone-treated counterparts.

To assess global disease activity, combined measures of animal body condition, pain, body weight, mobility and joint inflammation were collected. In TNFtg animals, a significant reduction in clinical scores was apparent, relative to vehicle-treated controls, in response to both prednisolone (PRED) and vamorolone (VAM) interventions (PRED, 18.1%, *P <* 0.01; VAM, 18.4%, *P <* 0.01) (Fig. 3E). However, in TNFtg^11BKO^ animals, whilst vamorolone continued to show a similar pattern with a significant decrease in clinical score (VAM, 41.4%, *P <* 0.001), the effects of prednisolone were largely blunted (Fig. 3F). Both prednisolone and vamorolone were effective at suppressing local joint inflammation scores relative to vehicle-treated controls (PRED, 48.6%, *P <* 0.001; VAM, 66.2%, *P <* 0.001) (Fig. 3G). Whilst similar trends were apparent in TNFtg^11BKO^ animals, decreases relative to vehicle-treated controls for both interventions were not significant [PRED, 28.4%, not significant (NS); VAM, 25.9%, NS], suggesting that neither intervention was suppressing disease activity effectively (Fig. 3H). Direct comparison of joint inflammation between TNFtg and TNFtg^11BKO^ animals revealed that scores were significantly greater at day 56 in vehicle-treated TNFtg^11BKO^ animals relative to TNFtg counterparts (TNFtg 6.8 ± 0.82 *vs* TNFtg^11BKO^ 8.1 ± 0.78, *P <* 0.05), suggesting that greater basal inflammation in the latter may require increased steroid dose to achieve effective anti-inflammatory efficacy.

Patterns of *Tnfa* and *Il-6* gene expression in synovial tissues from these groups revealed similar patterns, where prednisolone and vamorolone favoured more effective suppression of pro-inflammatory gene expression in TNFtg animals (*Tnfa*: PRED, 3.2-fold, *P <* 0.01; VAM, 1.4-fold, NS; *Il6*: PRED, 7.6-fold, *P <* 0.001; VAM, 4.5 fold, *P <* 0.001), but failed to demonstrate significant suppression in TNFtg^11BKO^ counterparts (Fig 3I and J). Similar trends were evident when circulating levels of IL-6 were measured (Fig. 3K). A marked suppression of IL-6 in the TNFtg cohort in response to prednisolone and vamorolone (PRED, 90.06%, *P <* 0.001; VAM, 72.2%, *P <* 0.05), was less evident in TNFtg^11BKO^ animals, despite demonstrating a similar trend (Fig. 3K).These data suggest that vamorolone is effective at reducing clinical scores and joint inflammation to a similar extent as prednisolone, but without eliciting the same degree of adrenal atrophy or systemic weight loss.

### Suppression of pannus formation and trabecular bone loss remain evident with vamorolone

**Figure 4.**
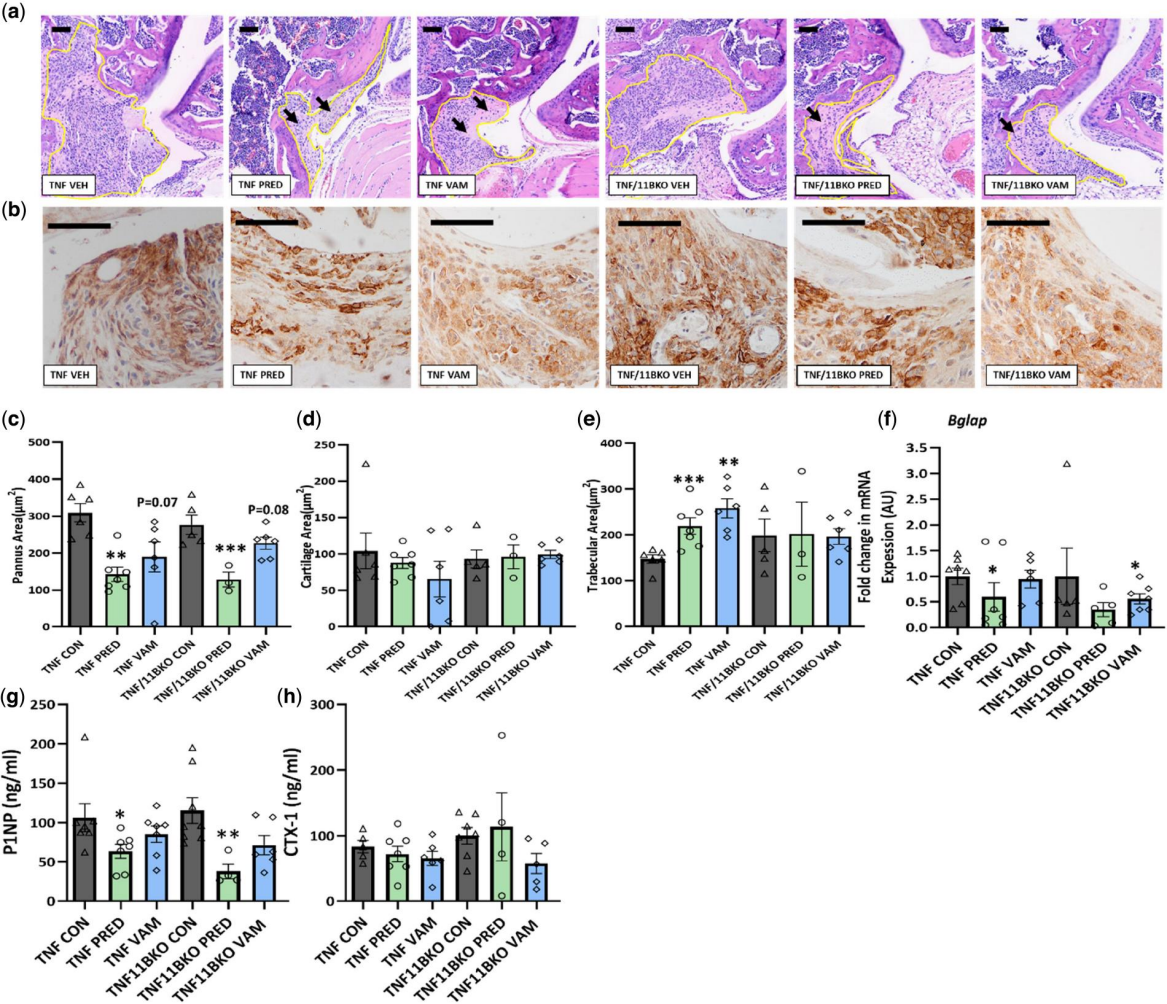
Suppression of pannus formation and trabecular bone loss remain evident with vamorolone. (**A**) Representative sagittal images of formalin-fixed paraffin-embedded elbow joints, stained with haematoxylin and eosin and (**B**) representative images of formalin-fixed paraffin embedded elbow joints analysed by immunohistochemistry for F4/80 protein (**C**) measurement sub-chondral pannus formation, (**D**) surface cartilage area and (**E**) trabecular area in TNFtg and TNF/11BKO animals at day 22 following treatment with oral gavage at 20 mg/kg of either prednisolone (PRED) and vamorolone (VBP15). Areas were measured using the ImageJ software. (**F**) *Bglap* gene expression measured in the femur following treatment with oral gavage at 20 mg/kg of either prednisolone (PRED) and vamorolone (VBP15) in TNF and TNF/11BKO mice for 21 days. Serum measures of (**G**) bone formation (P1NP) and (**H**) bone resorption (CTX1) were assessed by ELIZA in TNFtg and TNF/11BKO animals at day 22 following treatment with oral gavage at 20 mg/kg of either prednisolone (PRED) and vamorolone (VBP15). Black arrows denote positively affected areas of the joint. Data are presented as mean ± s.e.m. of at least six animals per group. Statistical significance was determined using one-way ANOVA with Tukey’s multiple comparisons test (*P ≤ 0.05, **P ≤ 0.01, ***P ≤ 0.001) (scale bars, 50 μm)

Histological analysis of formalin-fixed tissue sections was used to quantify changes in local joint destruction, pannus formation, cartilage erosion and juxta-articular trabecular loss in response to prednisolone and vamorolone in TNFtg and TNFtg^11BKO^ animals. In both TNFtg and TNFtg^11BKO^ animals, pannus area was significantly suppressed in response to prednisolone relative to vehicle-treated animals (TNFtg, 50.04%, *P <* 0.01; TNFtg^11BKO^, 46.11%, *P <* 0.001) (Fig. 4A and C). Whilst similar trends were apparent in vamorolone-treated animals, these both fell short of statistical significance (TNFtg, 43.87%, *P =* 0.07; TNFtg^11BKO^, 19.04%, *P =* 0.08) (Fig. 4A and C). Whilst the leucocyte infiltrate appeared to be more diffuse within the pannus of prednisolone and vamorolone treated animals, examination of the F480^+ve^ myeloid infiltrate did not reveal a marked change in the pattern of staining within the residual pannus relative to vehicle-treated animals (Fig. 4B).

Cartilage area remained unchanged between both TNFtg and TNFtg^11BKO^ animals and did not significantly change with either prednisolone or vamorolone interventions (Fig. 4D). In contrast, both prednisolone and vamorolone significantly increased sub-chondral trabecula area relative to vehicle treatment in TNFtg animals (PRED, 32.9%, *P <* 0.001; VAM, 42.98%, *P <* 0.001) (Fig. 4E). This protective effect was not evident following treatment with either prednisolone or vamorolone in TNFtg^11BKO^ animals. Of note, in both TNFtg and TNFtg^11BKO^ groups, treatment with prednisolone, but not vamorolone, significantly suppressed gene expression of the mature osteoblast marker *bone Bglap* (Fig. 4F). Further systemic measures of osteoblastic bone formation (P1NP) and osteoclastic bone resorption (CTX-1) were assessed across all groups (Fig. 4G and H). Serum P1NP levels closely mirrored patterns of *Bglap* expression, with a significant decrease in TNFtg and TNFtg^11BKO^ groups treated with prednisolone (TNFtg, 41.6%, *P <* 0.05; TNFtg^11BKO^, 68.9%, *P <* 0.01) (Fig. 4G). However, this suppression of P1NP was partially ameliorated in both groups after treatment with vamorolone. In contrast, changes in CTX1 as a measure of systemic bone resorption showing no significant change across the intervention wings (Fig. 4H). These data indicate that vamorolone has a similar profile of joint protective actions to that seen with prednisolone *in vivo*, suppressing pannus formation and increasing local subchondral trabecular bone, without suppressing measures of systemic osteoblastic bone formation to the same extent as prednisolone.

### Vamorolone does not drive steroid-induced muscle wasting at anti-inflammatory doses

**Figure 5.**
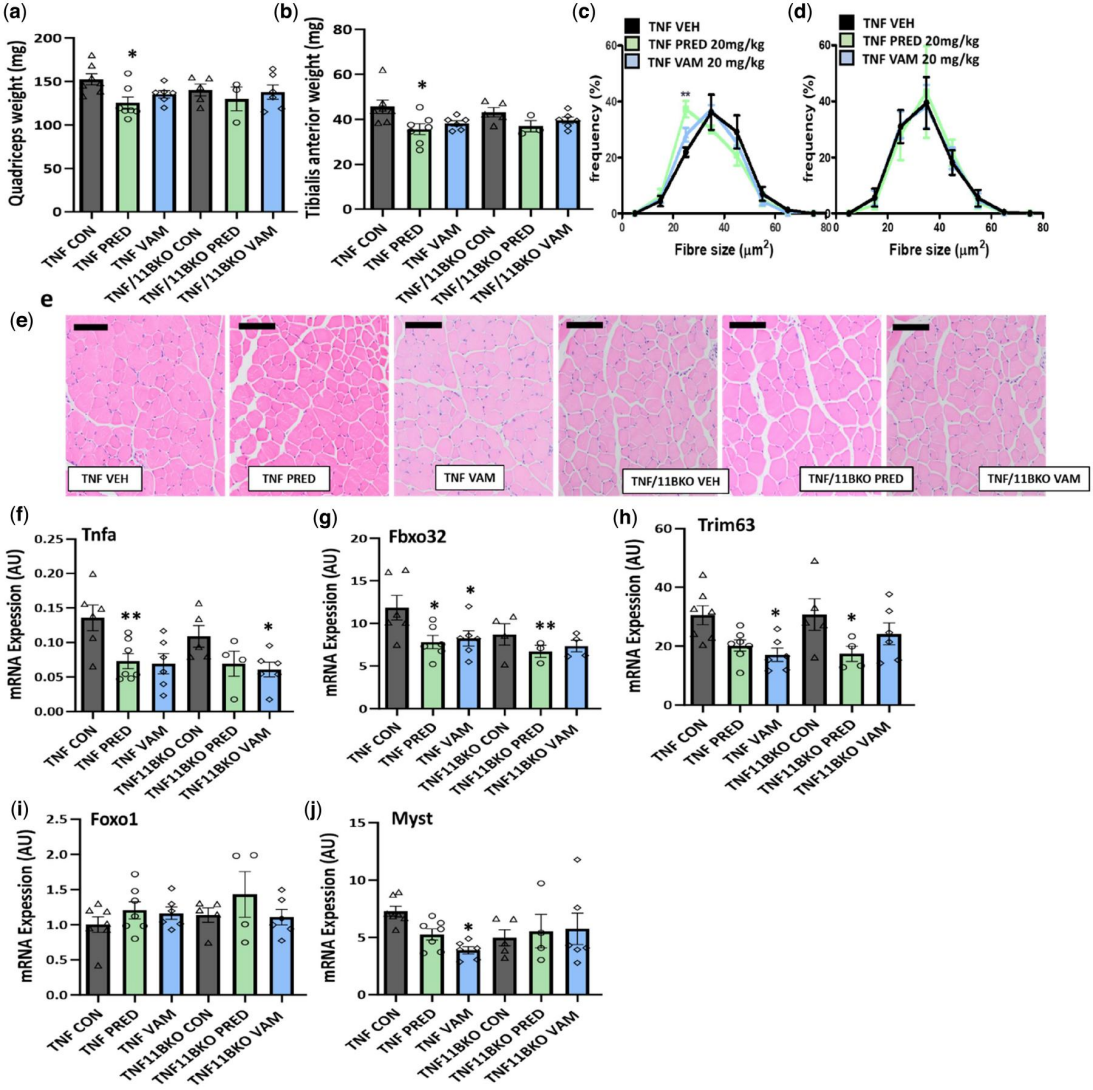
Vamorolone does not drive steroid-induced muscle wasting at anti-inflammatory doses. (**A**) Quadriceps weight and (**B**) tibialis anterior weight measured in TNF and TNF/11BKO mice at day 22 following treatment with oral gavage at 20 mg/kg of either prednisolone (PRED) or vamorolone (VBP15). Fibre size distribution measured in the quadriceps using ImageJ software (**C**) in TNF mice and (**D**) in TNF/11BKO mice at day 22 following treatment with oral gavage at 20 mg/kg of either prednisolone (PRED) and vamorolone (VBP15). (**E**) Representative images of formalin-fixed paraffin-embedded quadriceps sections of TNF and TNF/11BKO mice at day 22 following treatment with oral gavage at 20 mg/kg of either prednisolone (PRED) or vamorolone (VBP15). (**F**) *TNFa*, (**G**) *Fbxo32*, (**H**) *Trim63* (**I**) *Foxo1* and (**J**) *Myst* gene expression measure in the quad of TNF and TNF/11BKO mice at day 22 following treatment with oral gavage at 20 mg/kg of either prednisolone (PRED) or vamorolone (VBP15). Data are presented as mean ± s.e.m. of at least three six animals per group. Statistical significance was determined using one-way ANOVA with Tukey’s multiple comparisons test (*P ≤ 0.05, **P ≤ 0.01, ***P ≤ 0.001) (scale bars, 50 μm)

To assess the off-target actions of vamorolone in skeletal muscle, its effects on muscle weight and muscle fibre distribution of the quadriceps and tibialis anterior were measured relative to prednisolone. TNFtg animals receiving prednisolone exhibited a significant reduction in both quadriceps and tibialis anterior muscle weights relative to vehicle-treated controls (Quad, 12.05%, *P <* 0.05; TA, 22.61%, *P <* 0.05) (Fig. 5A and B). Whilst similar trends were apparent in TNFtg animals receiving vamorolone, these were less marked and not statistically significant. These changes were not apparent in the quadriceps of TNFtg^11BKO^ animals for either prednisolone or vamorolone, whilst a similar trend as seen in TNFtg animals was evident in tibialis anterior (Fig. 5A and B). Analysis of quadriceps fibre size indicated that TNFtg animals receiving prednisolone had an increased frequency of smaller fibres between 20 and 30 µm (41.11%, *P <* 0.01), whilst animals receiving vamorolone did not significantly differ (Fig. 5C and E). In contrast, treatment with either prednisolone or vamorolone did not alter fibre size in TNFtg^11BKO^ animals (Fig. 5D and E).

Gene expression of markers of muscle inflammation (*Tnfa*), catabolic signalling (*FBxo32*, *Trim63* and *Foxo1*) and anti-anabolic signalling (*Myst*) were examined in quadriceps from all groups (Fig. 5F–I). Both prednisolone and vamorolone favoured a modest, but comparable reduction in the expression of *Tnfa* in muscle from all TNFtg and TNFtg^11BKO^ animals (TNFtg: PRED, 1.64-fold, *P <* 0.01; VAM, 1.86-fold, NS; TNFtg^11BKO^: PRED, 1.56-fold, NS; VAM, 1.815-fold, *P <* 0.05) (Fig. 5F). Similarly, prednisolone and vamorolone suppressed the expression of *Fbxo32* and *Trim63* in TNFtg animals, whilst prednisolone, but not vamorolone, suppressed expression in TNFtg^11BKO^ animals (TNFtg *Fbxo32*: PRED, 1.95-fold, *P <* 0.05; VAM, 1.68-fold, *P <* 0.05; TNFtg^11BKO^: PRED, 1.58-fold, *P <* 0.01; TNFtg *Trim63*: PRED, 1.51-fold, *P =* 0.07; VAM, 1.76-fold, *P <* 0.05; TNFtg^11BKO^: PRED, 1.8-fold, *P <* 0.05) (Fig. 5G and H). In contrast, Foxo1 did not show evidence of regulation by prednisolone or vamorolone, whilst the anti-anabolic gene Myst was only downregulated in TNFtg animals receiving vamorolone (1.89-fold, *P <* 0.05) (Fig. 5I). Together these data suggest that vamorolone does not induce glucocorticoid-mediated muscle wasting within the therapeutic dosing utilized in this study, whilst suppressing markers of muscle inflammation and muscle catabolism.

## Discussion

Therapeutic steroids such as prednisone are effective at controlling inflammation in diseases such as RA but their use is limited by an array of severe off-target side effects. Vamorolone is a novel dissociated steroid that has shown efficacy in the treatment of Duchenne muscular dystrophy, without eliciting classic glucocorticoid-related side effects [10–12]. Its double bond between C9 and C11 prevents vamorolone from inducing classical homodimer glucocorticoid rsponse element (GRE)-mediated transactivation, but retains actions mediated by the ligand bound glucocorticoid receptor (GR) monomer [13, 15, 25]. This change impacts on the moiety involved in metabolism by the 11β-HSD1 enzyme. This enzyme is integral in mediating many positive and deleterious features of therapeutic glucocorticoids *in vivo*, amplifying their levels in tissues such as adipose, muscle and bone. We used the well-characterized murine TNFtg model of chronic polyarthritis with global transgenic deletion of 11β-HSD1 to explore whether the resistance of vamorolone to this enzyme contributed to its improved profile of side effects.

The metabolism of vamorolone by 11β-HSD1 was characterized using a well-established *in vitro* cell system. Vamorolone was completely resistant to metabolism by the enzyme 11β-HSD1, in keeping with its modified structure, lacking the functional ketone and hydroxyl groups at C11 [14, 18]. In the TNFtg model of polyarthritis, vamorolone possessed an equivalent immunomodulatory action as prednisolone. This was characterized by the suppression of disease activity, as well as systemic pro-inflammatory mediators such as IL-6 in the serum, and potent inflammatory mediators such as TNF-α in tissues such as muscle, underpinning elements of it therapeutic efficacy in other disease settings such as Duchenne muscular dystrophy [26]. However, as opposed to prednisolone, animals receiving vamorolone were protected from common deleterious side effects of prednisolone, including adrenal atrophy, weight loss and muscle wasting. These data are in keeping with the well-established status of vamorolone as a dissociated steroid, however aspects of vamorolone action on expression of the classically transactivated gene *Gilz* were evident in primary macrophages [13, 15, 25]. Whilst this study was not able to address the exact mechanistic processing underpinning this, we were able to show that this was not occurring secondary to metabolism to prednisolone *in vitro* or *in vivo* by LC-MS over the collection points examined. It may be that over an extended timeframe, further metabolic processes may drive this nature of metabolism. Alternatively, the GR-mediated non-genomic suppression of factors such as p38-MAPK (that have been shown to positively regulate Gilz and enzymes that mobilize endogenous glucocorticoids) may indirectly upregulate Gilz expression independently of transactivation [27–29]. In this study, several of the effects of prednisolone were also dependent on 11β-HSD1, where suppression of disease scores and muscle weights were abrogated in animals with transgenic deletion of 11β-HSD1 as previously reported [6, 19]. In contrast, vamorolone retained its ability to influence these measures in TNFtg^11BKO^ animals. Other actions of vamorolone and prednisolone in the TNFtg^11BKO^ group were partially attenuated altogether. These data appear to reflect an increased level of inflammatory disease burden in TNFtg^11BKO^ animals, where both disease scores and inflammatory paw scores were elevated relative to TNFtg counterparts. Indeed, we have previously shown that these animals experience an increased disease severity because of an inability to activate endogenous glucocorticoids by 11β-HSD1 at sites of inflammation [26].

Consequently, the increased disease burden in TNFtg^11BKO^ animals may necessitate a greater dose of steroid to suppress disease than used in this study (20 mg/kg). This would mirror higher doses of vamorolone, at 40 mg/kg, required to suppress inflammation in the acute inflammatory CIA model of polyarthritis [16]. In this study, we wished to examine the direct actions of both prednisone and vamorolone at matched doses to ensure equivalent modelling of local enzyme kinetics. Other studies reported that effective dosing of vamorolone requires roughly four times that of prednisolone to achieve equivalent immune suppression [16, 30]. These observations are most evident in models where immunological adjuvants such as lipopolysaccharide and pertussis toxin are utilized [16, 30]. In contrast, vamorolone and prednisolone possess comparable anti-inflammatory action in models of disease that are independent of adjuvant induction [13, 14, 18]. The TNFtg model of chronic polyarthritis is an adjuvant-independent model of disease, and the matched anti-inflammatory response to both vamorolone and prednisolone at 20 mg/kg support these latter studies. Adjuvants such as lipopolysaccharide potently induce 11β-HSD1 within leukocytes populations such as macrophages and may explain why locally metabolized steroids such as prednisolone have increased efficacy relative to metabolism resistant formulations such as vamorolone [27, 31, 32].

Many studies have indicated that 11β-HSD1 is upregulation within off-target tissues such as muscle and bone in chronic inflammatory diseases and with advancing age, where they drive adverse musculoskeletal side effects of therapeutic glucocorticoids [6, 19, 20, 23, 33, 34]. This is pertinent the resistance to metabolism by may contribute to its reduced profile of musculoskeletal side effects in this study. At therapeutic doses, steroids such as prednisolone potently suppress markers of anabolic bone formation (such as P1NP, osteocalcin and alkaline phosphatase) within bone, driving a progressive glucocorticoid-induced osteoporosis [35]. Not only did vamorolone avoid this deleterious side effect of traditional glucocorticoids in bone, preserving *Bglap* expression and serum P1NP levels, it also protected against inflammatory trabecular bone loss adjacent to arthritic joints in a similar manner to that of prednisolone [36]. Consequently, it appears that the actions of vamorolone may be dissociated in a fashion that favours the protective anti-resorptive actions without driving a wider suppression of anabolic bone formation in this setting. Further measures of systemic bone metabolism in long bones and spine, using measures such as micro-CT, and static and dynamic histomorphometry are now required to comprehensively characterize this phenotype. Recent data from human studies, where vamorolone is administered to boys with Duchenne muscular dystrophy, support the relevance of these bone metabolism observations [9].

## Conclusions

This study reveals that vamorolone possesses comparable anti-inflammatory efficacy as that of prednisolone in the TNFtg model of polyarthritis, with a reduced profile of undesirable effects. These actions were largely independent of 11β-HSD1 metabolism. Further research is now required to understand and delineate in what inflammatory disease settings this novel dissociated steroidal compound can be applied in the clinic.

## Data Availability

The authors confirm that the data supporting the findings of this study are either available within the article or available from the corresponding author on request.
